# Exclusion-zone water inside and outside of plant xylem vessels

**DOI:** 10.1038/s41598-024-62983-3

**Published:** 2024-05-27

**Authors:** Anqi Wang, Gerald H. Pollack

**Affiliations:** Department of Bioengineering, University of Washington, Box 355061, Seattle, WA 98195 USA

**Keywords:** Plant physiology, Colloids

## Abstract

The fourth phase of water has garnered increased attention within the scientific community due to its distinct properties that differentiate it from regular water. This unique state seems to arise potentially from a liquid crystalline structure, which has been observed near various hydrophilic surfaces to possess the capability of excluding microspheres. Consequently, it has been labeled as exclusion zone (EZ) water. When in contact with hydrophilic surfaces, water could exhibit the ability to form organized layers of EZ water. In this study, we investigated the quick buildup of EZ water exposed to xylem vessels of four vegetable plants: cabbage, celery, asparagus, and pumpkin. Among them, pumpkin vessels showed larger EZs, up to 240 ± 56 μm in width. The width of EZ water found near the xylem vessels of the other plants ranged from 133 ± 22 to 142 ± 20 μm. EZ water generally excludes a wide range of particles, including polystyrene microspheres with various surface modifications, as well as silica microspheres. This implies that the formation of EZ water is not an artificial result of using specific microsphere types but rather demonstrates EZ’s ability to exclude particles regardless of their composition. Inside single xylem vessels of the pumpkin, we could observe the dynamics of EZ buildup, growing from the inside edge of the vessel toward the center. The relationship between vessel diameter, vessel length, and salt concentration on EZ generation inside the xylem vessel was also explored. The results showed that EZ water can build up both inside and outside the xylem vessels. Our findings suggest that EZ generation inside xylem vessels is associated with water flow, likely driven by a proton gradient. Further research is warranted to elucidate the role of EZ water in the physiology of living plants, particularly considering the limitations of the current experiments conducted on cut-out xylem vessel samples.

## Introduction

Seeing a giant redwood tree more than 100 m tall inevitably raises the question of the driving force responsible for lifting the water to such great heights. For more than 100 years, the ascent of sap has been ascribed to a “cohesion-tension” theory (CTT). According to this theory, water is pulled up through the xylem conduit by tension gradients produced by water loss during evaporation^1–4^. While the CTT provides a fundamental framework for understanding plant water transport, certain aspects remain contentious.

Therefore, various hypotheses have been proposed, suggesting the involvement of multiple forces in the process of water ascent, including osmotic pressure gradients, electrical potential gradients, and interfacial gradients^5–7^. While alternative theories haven't replaced the CTT, they've prompted broader research into xylem water transport. Xylem water movement involves complex interfacial effects, increasingly recognized as crucial for understanding plant-water dynamics. In our study, we investigate a particle-excluding water domain within xylem vessels and the localized flow it induces.

A water region capable of excluding particles has been found on the surfaces of various hydrophilic materials. Those surfaces include Nafion^8–13^, hydrogels^14–16^, cellulose^17^, metals^18^, clay^19^, silk sericin^20^, propolis^21^, and muscle^14,15^. This water region, capable of excluding solutes and particles up to several hundred micrometers wide, is also known as exclusion zone (EZ) water. Further studies on EZ water have revealed some unique characteristics distinct from ordinary liquid water, including a higher refractive index and viscosity, as well as light absorption at 270 nm^22^. It is thus referred to as “the fourth phase of water.” Another distinctive feature of this water is its charge. Tiny electrodes have detected electrical potential as large as − 200 mV in the EZ^23^, indicating negative charges within the EZ, whereas the surrounding water zone carries a positive charge due to hydronium ions^24^.

EZ water plays an important role in the generation of a surface-induced water flow in Nafion tubes as well as in various hydrophilic channels^16,25,26^. During EZ buildup, protons leave the EZ, concentrating beyond the EZ’s far boundary. As positive charges accumulate within the tube, a proton gradient forms (positive charge inside the tube core and zero charge outside the tube ends), driving the positive charges out of the open ends. This gradient can induce water flow in either direction, depending on asymmetries in the system. Once flow starts in one direction, water is drawn into the tube at the other end to replace displaced water, perpetuating the flow^16,26,27^. As water flows through the tube, it acquires protons, increasing the concentration along the tube's length and maintaining the proton gradient. Eventually, flow may stop as protons accumulate, making it more difficult to establish gradients. The energy source for the flow appears to be infrared (IR) energy, as IR is an efficient builder of the EZ^24,28–31^.

Evidence exists that plant xylem may contain EZ water. First, the major components of the xylem cell walls are cellulose, hemicellulose, and lignin. The hydrophilic cellulose surface has been reported to be prone to generating EZ water^17^. Further, xylem sap is generally acidic, which is consistent with the evidence that EZ water releases positively charged hydronium ions^14,23,24^. Given the apparently close association of xylem vessels with EZ water, we aimed to seek direct evidence for the presence of EZ water in plant vessels, and to study the factors influencing EZ-water generation. Four plant species were selected for this study, including celery (*Apium graveolens*), napa cabbage (*Brassica rapa* subsp. *pekinensis*), asparagus (*Asparagus officinalis* L.) and pumpkin (*Cucurbita pepo* L.). These species were chosen because of their large vessel diameter and ready availability. Finally, we investigated the possibility that EZ water inside vessels drives a flow.

**Figure 1:**
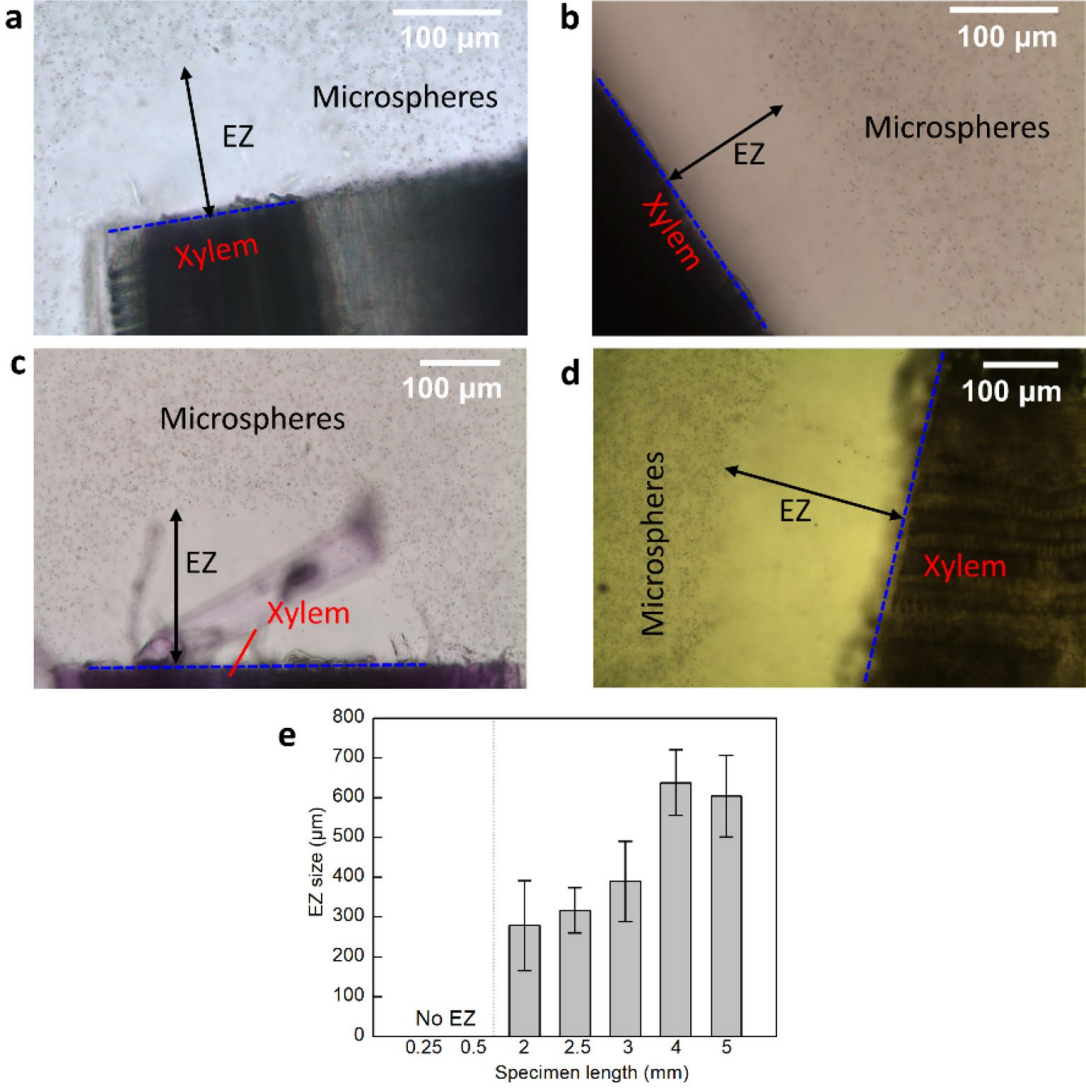
EZ water formed at the end of (**a**) napa cabbage, (**b**) celery, (**c**) asparagus, and (**d**) pumpkin vessel. The blue lines denote the ends of the vessels. (**e**) Effect of vessel specimen length (in the longitudinal direction) on size of the EZ outside xylem vessels (n = 5; error bars denote SD).

EZ water began building up immediately at both ends of the immersed vessels, progressively excluding the microspheres. The size of the EZ, representing its width, stabilized after approximately 5 min. As shown in Fig. 1a–c, the width of EZ water found near the xylem vessel of napa cabbage, celery, and asparagus (n = 9) ranged from 133 ± 22 to 142 ± 20 μm. In Fig. 1d, the width of EZ water adjacent to the xylem vessel of the pumpkin (n = 9) was as large as 240 ± 56 μm. We measured the inner diameter of single xylem vessels (n = 10) for each type of plant, and the results showed that the average diameter of pumpkin vessel (84 ± 33 μm) was larger than that of napa cabbage (27 ± 6 μm), celery (30 ± 7 μm), and asparagus (35 ± 9 μm). The effect of vessel length on EZ width outside xylem vessels was shown in Fig. 1e. EZ size increased with increasing vessel length. The largest EZ size (637 ± 82 μm) was achieved at a length of 4 mm or higher. To clarify the effect of vessel length on EZ size, it is important to understand how the EZ is formed. The first layer of structured water molecules forms at the hydrophilic surface. Subsequent layers then stack onto that layer to build up the EZ^32^. Since longer vessels had a larger surface area onto which EZ could build up, they were likely to form larger EZs. In Fig. 1e, no EZ was observed in short vessels (< 0.5 mm). This finding suggests that the initiation of EZs from hydrophilic surfaces could be hindered in shorter vessels due to insufficient surface area.

The formation of EZ water outside xylem vessels was tested using different microsphere types: carboxylate, polystyrene, amino, and silica. Carrying different surface groups, these microspheres hold different charges: carboxylate and polystyrene have negative charge, amino positive, and silica microspheres have no charge. Despite these varying charges, EZ water was formed consistently, in all samples (n = 3 each).

**Figure 2:**
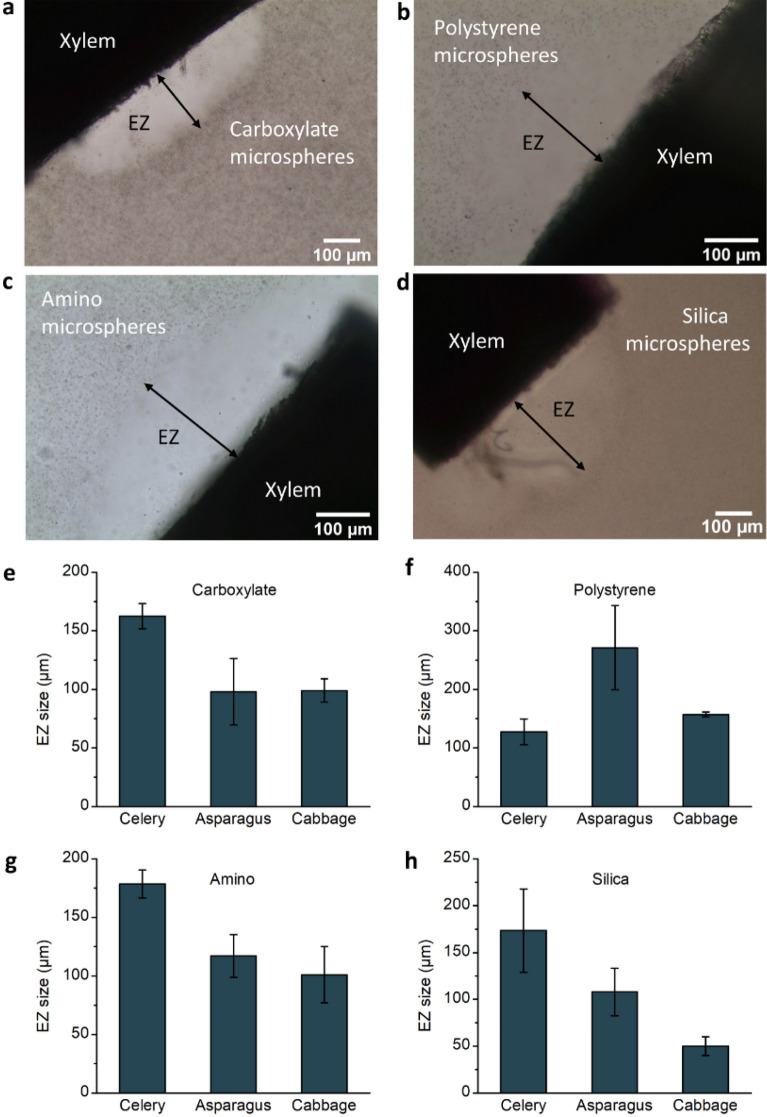
Effect of microsphere suspension on EZ water. EZ water typically formed next to the ends of celery vessels in different types of microsphere suspension, including (**a**) carboxylate, (**b**) polystyrene, (**c**) amino, and (**d**) silica. (**e**–**h**) EZ size outside xylem vessels in different types of microsphere suspensions (n = 3; error bars denote SD).

We found clear EZ boundaries in the carboxylate suspensions (Fig. 2a). Carboxylate microspheres may release H^+^ due to the ionization of surface carboxyl groups. On the contrary, EZ water is negatively charged; therefore, because of the attraction of opposite charges, some carboxylate microspheres might aggregate at the edge of the EZ, setting up a clear boundary. Silica microspheres also slightly accumulated at the edge of the EZ (Fig. 2d). Polystyrene and amino microspheres (Fig. 2b, c) did not aggregate at the boundary; instead, they dispersed sparsely in the bulk water. This phenomenon may be attributed to the ability of these microspheres to generate OH^−^ ions through hydrolysis, which are of the same charge as those present in EZ water. We measured the size of EZ formed outside the vessel in different microsphere suspensions (Fig. 2e–h). EZ size differed among both plant species and particle type. Except for asparagus, which shows the largest EZ size with polystyrene particles, celery vessels tended to produce the largest EZ with the other three kinds of microspheres. One reason might be that celery has more xylem vessels with hydrophilic surfaces than asparagus and cabbage (Fig. S2).

**Figure 3:**
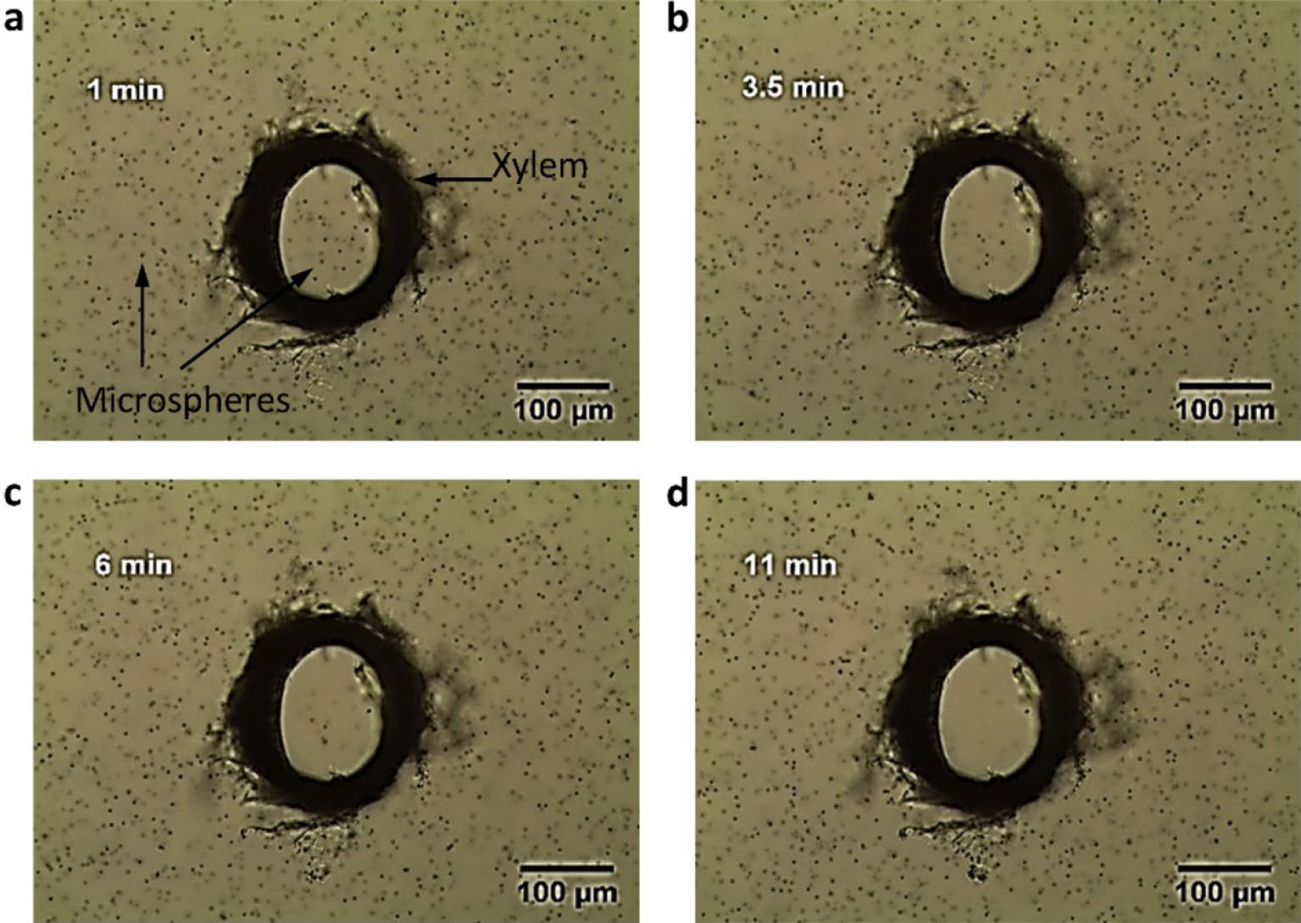
Time course of EZ buildup inside a single pumpkin xylem vessel at (**a**) 1 min, (**b**) 3.5 min, (**c**) 6 min, and (**d**) 11 min. By using a dissection needle and a tweezer, we could isolate and observe single pumpkin vessels with a length of 200 μm and an inner diameter of ~ 230 μm from a transverse perspective. The isolated vessel was carefully placed vertically into a suspension of carboxylate microspheres.

Having observed EZ outside the xylem vessels in four plants, we turned to address the situation inside the vessels, made challenging because of vessel opacity. To pursue this question, we explored pumpkin xylem vessel. Because the inner diameters of the pumpkin vessels are as large as 100–200 μm, it was feasible to explore single vessels. Figure 3 shows representative images of EZ formation over time. When the vessel was initially immersed in the carboxylate suspension, the microspheres were uniformly distributed both inside and outside the vessel (Fig. 3a). With increasing time, although the microspheres outside the vessel remained dispersed, the inner microspheres began to migrate towards the center of the vessel lumen, leaving a microsphere-free zone near the inner wall (Fig. 3b, c). Carbon-coated iron nanoparticles were reported to be similarly migrated towards the center inside the xylem vessels of pumpkin^32^. By 11 min, almost all microspheres were excluded from the inside of the vessel (Fig. 3d), apparently replaced by EZ water. We need to clarify that the observations here are made from a transverse perspective, in which no EZ is observed outside the vessel. The EZ in Figs. 1 and 2 refers to what is observed at the ends of the vessel from a longitudinal perspective, which can last for more than 20 min.

We then explored the effect of salt (NaCl) concentration, vessel diameter and vessel length on EZ buildup. We measured the size of EZ inside the single vessel in a transverse perspective at the midpoint along the vessel’s length. The total area inside the vessel (A_T_) can be divided into two parts, the EZ-containing area (A_EZ_) and the aggregated microsphere area (A_M_). The respective areas can be measured by using the software ImageJ. The fractional EZ area ratio was calculated by dividing A_EZ_ by A_T_, as shown in Eq. (1).

1 $$ EZ\; area\; ratio = \frac{{A_{EZ} }}{{A_{T} }} \times 100\% = \frac{{A_{T} - A_{M} }}{{A_{T} }} \times 100\% $$

**Figure 4:**
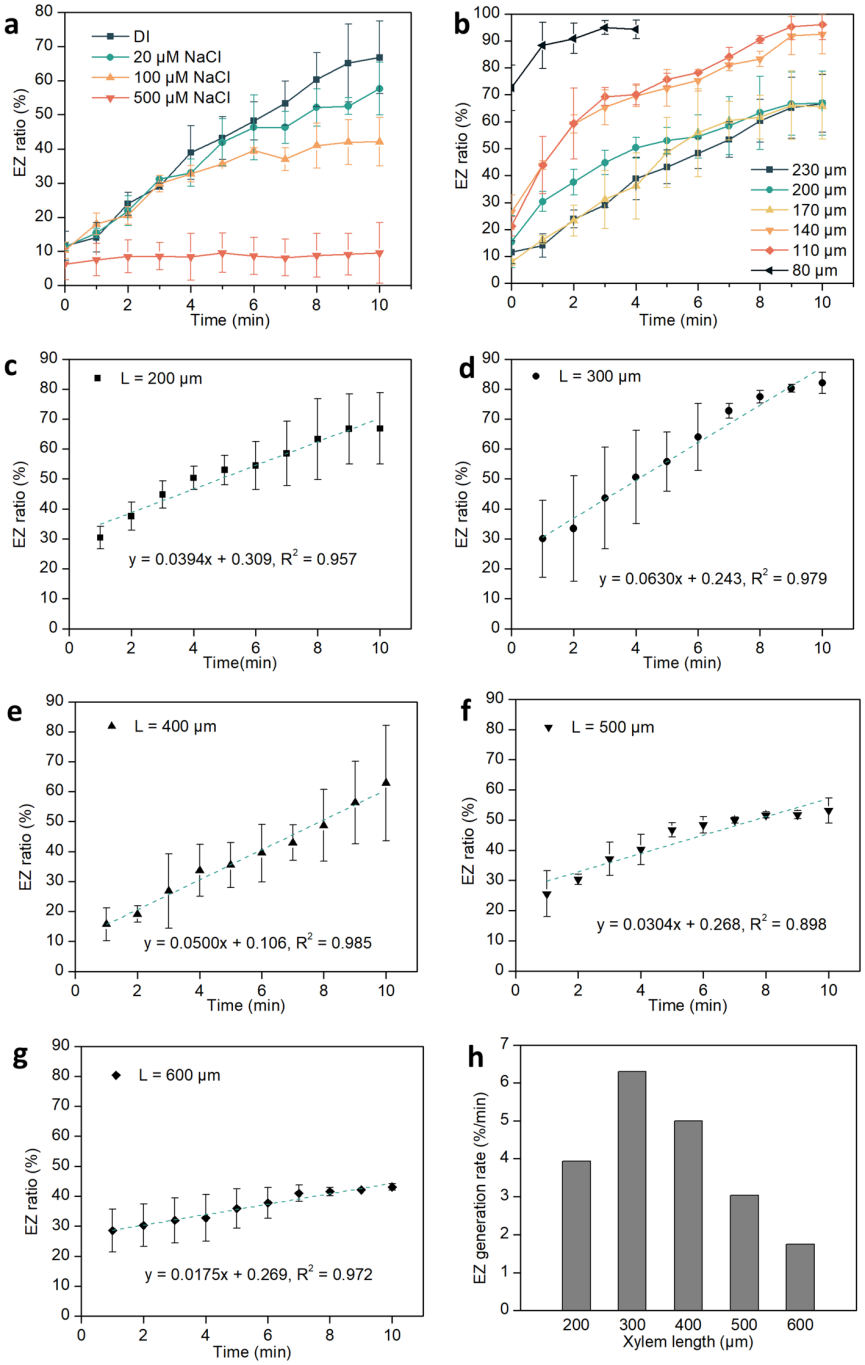
Time course of EZ-area ratio in single pumpkin xylem vessels. Effect of (**a**) salt concentration, (**b**) vessel diameter, and (**c**–**h**) vessel length on EZ development. In (**a**–**g**), n = 3; error bars denote SD.

Figure 4a shows that the EZ ratio decreased as the salt concentration increased from 0 to 500 μM. When the salt concentration was less than 100 μM, an EZ fraction of at least 40% could be reached in 10 min. On the other hand, when the concentration of salt was 500 μM, the fraction was less than 10%, which means only little EZ was generated. A similar drop in EZ size with increasing salt concentration was observed in previous studies^12,23^. In Fig. 4b, it is observed that a smaller vessel diameter correlates with a quicker filling of the vessel lumen. In vessel lumen diameter of 80 μm, EZ built up quickly. At 4 min, the EZ area reached the maximum of 94 ± 3% of the cross-sectional area, indicating that the interior of the vessel was filled with EZ water. With vessel diameter of 110–140 μm, 10 min were required for EZ water to grow to the maximum. For diameters larger than 170 μm, the EZ-area ratio was only 66 ± 12% at 10 min, indicating that larger areas require more time for building up. The diameter of xylem vessels in different plants can vary considerably, ranging from 8 to 500 μm ^33^, of which our experiment covered a substantial fraction of that range.

We investigated the variation of the EZ-area ratio over time in specimens of same diameter (200 μm) but different length varying between 200 and 600 μm (Fig. 4c–h). Vessels shorter than 200 μm could be seriously affected by end effects, while those longer than 600 μm were difficult to set up vertically, so they were not included in this study. In each batch of experiments, the rates of EZ buildup were analyzed by linear regression, with the equations labeled in Fig. 4c–g. As shown in Fig. 4c–h, EZ generation was fastest at a xylem vessel length of 300 μm, with an EZ-area ratio of 82 ± 4% at 10 min. When the length exceeded 300 μm, the rate of EZ generation decreased with increasing length. With length of 600 µm, for example, the EZ ratio at 10 min was only 43 ± 1% (Fig. 4g, h). In Fig. 4h, EZ generation rate represents the regression coefficient in Fig. 4c–g, which describes how fast the EZ was formed. As shown in Fig. 4h, in longer xylem vessel, EZ built up more slowly.

In the experiment outlined above, the expansion of the EZ not only occurred but also triggered an upward water flow within the vertically oriented vessel. However, in the presence of high salt solution (500 μM), where EZ water formation was minimal, no observable evidence of flow was detected. Previous research has documented analogous flow phenomena within Nafion tubes and various cylindrical tunnels embedded within hydrophilic materials^16,25,26^. The flow appears to be associated with the formation of EZ water. As EZ water nucleates at the material’s surface, protons accumulate in the water exterior to the EZ^22,24^. The accumulation of protons, in the form of hydronium ions, easily creates a proton gradient along the tunnel, resulting in the surface-induced flow. The driving energy appears to come from light, particularly likely infrared light since IR helps build EZ water, which in turn creates the proton gradient^24,30^.

The water flow we observed in the xylem vessel may be based on the same principle. A possible driving force could be the vertical concentration gradient of protons inside and outside the vessel. As annular EZ water grows layer by layer at the inner surface of the vessel, protons are released into the core. In this way, protons build up inside the vessel, inevitably creating the vertical proton gradient. Those core protons may then flow out from the open upper end of the vessel, where less resistance exists, creating the upward water flow inside the vessel. Driven by this flow, bulk water enters the vessel from the lower portion, providing raw materials for further formation of the EZ inside the xylem vessel, which sustains the flow. As long as EZ continues to build, the flow can be sustained; we observed the flow persisting for at least 10 min.

The intriguing EZ water and water flow reported in this study may contribute to a deeper understanding of xylem physiology. These findings hold promise as an additional component in comprehending the complex interfacial effects within the xylem. Our research lays the groundwork for further in-depth and open-ended exploration, emphasizing the need for more research to elucidate the role of EZ water and EZ-induced water flow in living plants.

## Methods

### Materials

Fresh whole celery (*Apium graveolens*), whole napa cabbage (*Brassica rapa* subsp. *pekinensis*), and asparagus stalks (*Asparagus officinalis* L.) were obtained from local markets (Seattle, WA, USA). Pumpkin (*Cucurbita pepo* L.) stems were cut from field-grown pumpkin plants. All plants were immediately returned to the laboratory after collection and stored in the lab refrigerator at 4 ℃ in zip-lock bags before experimentation. Our research involving the collection of pumpkin stem samples comply with relevant institutional, national, and international guidelines and legislation, with all necessary permissions and licenses obtained to ensure compliance with ethical and legal requirements.

Deionized (DI) water (18.2 MΩ cm) was obtained from a water purification system (Nanopure Diamond, Barnstead). Toluidine blue O (TBO, ≥ 95.0%, J.T.Baker) was utilized for staining the vessel. NaCl (≥ 99.0%, Fisher Chemical Company) was used to study the effect of salt. Carboxylate, polystyrene, amino, and silica microsphere suspensions (1 μm, 2.5% w/v, Polysciences) were employed to observe the EZ. Carbon steel rib-back scalpel blades (# 21) and blade handle (Bard-Parker) were used to cut stems into sections. Surgical carbon steel razor blades (VWR) were utilized for cutting stem slices when employing a microtome (United Scientific). New scalpel and razor blades were used in each batch of experiments.

### Observation of EZ outside of xylem vessels

We utilized napa cabbage, celery, asparagus, and pumpkin for our experiments. In preliminary experiments, plant stems were cut into sections and submerged in an aqueous microsphere suspension for observation. We found that microspheres were excluded in the vicinity of xylem vessels at both ends of the plant stem. This exclusion appeared similar to previous observations of EZ built up next to various hydrophilic surfaces^8,9,16^.

After numerous repetitions of preliminary experiments, we determined that the quality of the stem cut significantly influenced EZ observations. A flat, clean-cut surface facilitated clear EZ observation next to xylem vessels, while rough cuts resulted in less distinct EZ edges. Additionally, the presence of a significant number of tissue fragments near the cut reduced the size of the EZ. Thus, flat, clean cuts were deemed crucial for accurate observation in these experiments.

Another practice we adopted to successfully observe vessel associated EZ water was to immerse the fresh stem in boiling DI water for 10 min, which made it easier to separate the vascular bundle from other tissues. The bundles were then cut to obtain clear cross-sections, reducing interference from debris of dermal tissue or ground tissue. Experiments carried out under both conditions, i.e., with or without boiling treatment, showed no significant difference in EZ sizes (Fig. S1, p > 0.05).

After boiling, vascular bundles were cut into 2-mm segments and pre-soaked in DI water before use. Sample pieces were then transferred by using forceps into 0.1% toluidine blue solution for 2 min of staining. That solution dyed the vessel’s lignin blue, allowing xylem vessels to be distinguished under the microscope. Figure S2 shows xylem vessels of three vegetables: napa cabbage, celery, and asparagus. After preliminary observation, the dye was washed out by soaking the samples in DI water for 5 min.

Following that procedure, a sample piece was transferred into a chamber containing 200 μl of a prepared microsphere suspension (1:300). A cover-glass slide was then set on the top of the chamber to prevent air-convection disturbance. The chamber was made from a 2-mm thick transparent plastic block with a hollow cylindroid (11 mm in diameter) cut out in the center. Glued to the bottom of the chamber was a transparent 1-mm thick glass slide. The chamber was carefully placed on the stage of a microscope (Axiovert 100TV, Zeiss), equipped with both a camera (EO-3112C, Edmund Optics) and two objective lenses (5× and 10×, Zeiss). The microscope was equipped with a halogen lamp as its light source. This setup facilitated the observation of microsphere-free zones. Microscopy images were analyzed by ImageJ. Carboxylate, polystyrene, amino, and silica microspheres were used. With this preparation, we could investigate the effect of xylem vessel length on EZ size.

### Observation of EZ inside of single isolated xylem vessel

For investigation of EZ inside xylem vessels, we used pumpkin stems. Their diameters were as large as 100–200 μm, so EZ buildup could be directly observed under the microscope. Stem sections about 4 cm in length were obtained through transverse cuts of the pumpkin stem, with 0.5 cm lengths at both ends cut off and discarded. The remaining stem was fixed lengthwise in a microtome. Next, transverse cuts with a thickness of 200 μm were made using a hand microtome and a razor blade. The samples were then immediately immersed in DI water.

Individual xylem vessels were dissected out from those sample pieces. First, the stem slice was observed in cross-sectional views under the microscope. Then, under microscopy, a tweezer and a dissection needle were manipulated simultaneously to peel a single xylem vessel out from the sample piece. A schematic diagram of the dissection process is shown in Fig. S3. After dissection, the lengths and diameters of intact single xylem vessels were measured under the microscope. During all of the above procedures, the vessel was continuously soaked in DI water. Then, a single vessel was transferred into a covered petri dish containing 3 ml of the microsphere suspension. The tweezer and dissecting needle were used to make quick adjustments to ensure vertical orientation. The microscope’s focal plane was immediately adjusted to lie in the middle of the vessel’s length to reduce any effects of water-flow disturbances at both ends of the vessel. The influence of vessel diameter (80–230 μm), vessel length (200–600 μm), and salt concentration (0–500 μM), on the formation of EZ water inside the xylem vessel was then investigated.

### Supplementary Information

Supplementary Figures.

## Data Availability

All data generated or analysed during this study are included in this published article [and its supplementary information files].
