# Supplementary material for: Exclusion-zone water inside and outside of plant xylem vessels
Source: Sci Rep. 2024 May 27;14:12071. doi: 10.1038/s41598-024-62983-3 (PMC11130298; doi:10.1038/s41598-024-62983-3)
Supplement: Supplementary file 1 — Supplementary Figures. [file 41598_2024_62983_MOESM1_ESM.docx]

**Supporting Information**

**Exclusion-zone water inside and outside of plant xylem vessels**

Anqi Wang^1,^* and Gerald H. Pollack^1^

^1^Department of Bioengineering, University of Washington, Box 355061, Seattle, Washington 98195, United States

*Corresponding author email: anqiw66@uw.edu

Total number of pages: 4

Total number of Figures: 3


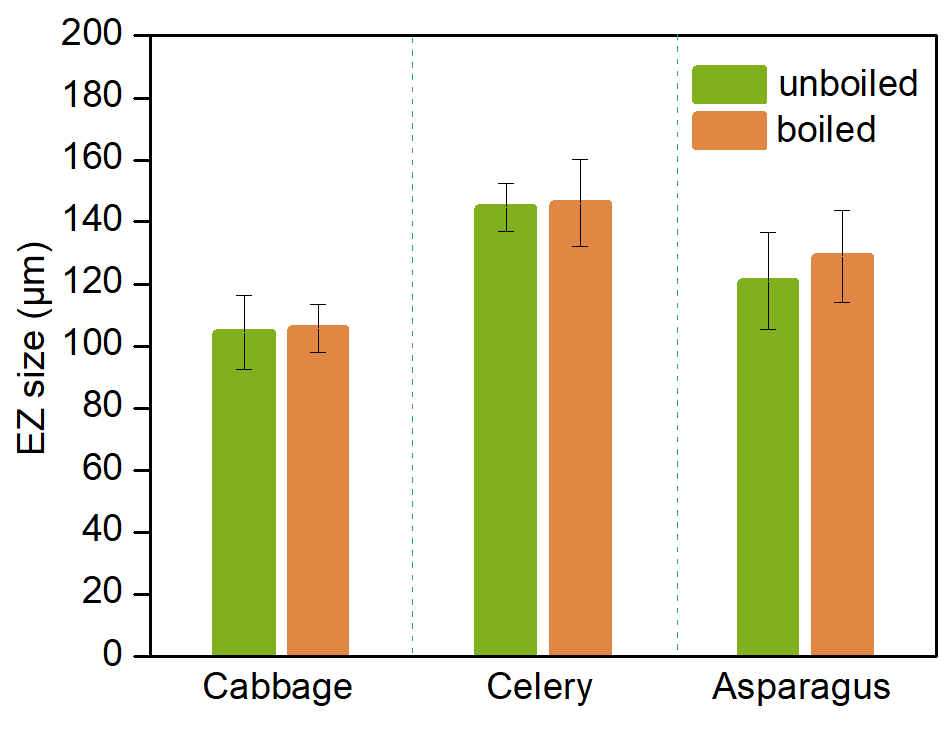


**Fig. S1.** EZ next to xylems with or without boiling treatment showed no significant difference (p > 0.05 for all three species).


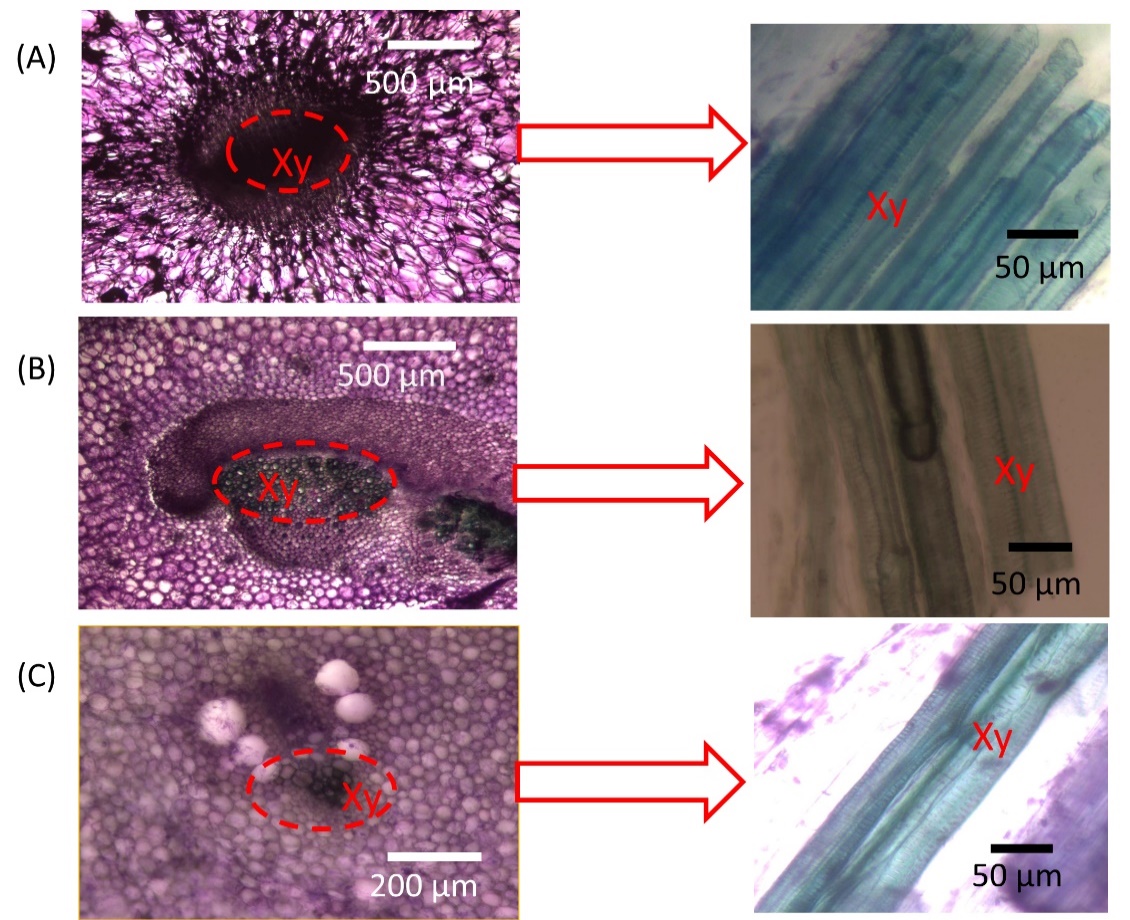


**Fig. S2.** Xylem vessels (dyed blue) of (A) napa cabbage, (B) celery, and (C) asparagus. The red label “Xy” represents for xylem.


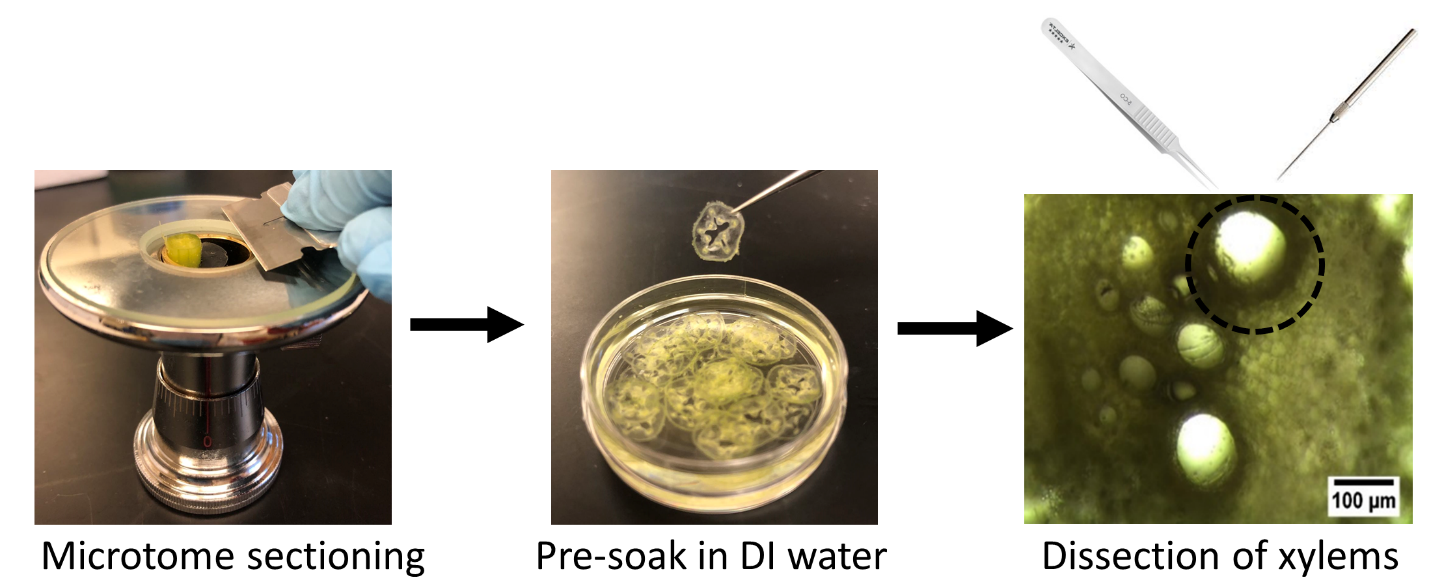


**Fig. S3.** Schematic diagram of the dissection process of the pumpkin xylem.
